# Fractal analysis of concurrently prepared latex rubber casts of the bronchial and vascular systems of the human lung

**DOI:** 10.1098/rsob.190249

**Published:** 2020-07-08

**Authors:** Montanna Essey, John N. Maina

**Affiliations:** Department of Zoology, University of Johannesburg, Auckland Park Campus, Kingsway, Johannesburg 2006, South Africa

**Keywords:** lung, branching, fractal geometry, fractal dimension, Hess–Murray law

## Abstract

Fractal geometry (FG) is a branch of mathematics that instructively characterizes structural complexity. Branched structures are ubiquitous in both the physical and the biological realms. Fractility has therefore been termed nature's design. The fractal properties of the bronchial (airway) system, the pulmonary artery and the pulmonary vein of the human lung generates large respiratory surface area that is crammed in the lung. Also, it permits the inhaled air to intimately approximate the pulmonary capillary blood across a very thin blood–gas barrier through which gas exchange to occur by diffusion. Here, the bronchial (airway) and vascular systems were simultaneously cast with latex rubber. After corrosion, the bronchial and vascular system casts were physically separated and cleared to expose the branches. The morphogenetic (Weibel's) ordering method was used to categorize the branches on which the diameters and the lengths, as well as the angles of bifurcation, were measured. The fractal dimensions (*D*_F_) were determined by plotting the total branch measurements against the mean branch diameters on double logarithmic coordinates (axes). The diameter-determined *D*_F_ values were 2.714 for the bronchial system, 2.882 for the pulmonary artery and 2.334 for the pulmonary vein while the respective values from lengths were 3.098, 3.916 and 4.041. The diameters yielded *D*_F_ values that were consistent with the properties of fractal structures (i.e. self-similarity and space-filling). The data obtained here compellingly suggest that the design of the bronchial system, the pulmonary artery and the pulmonary vein of the human lung functionally comply with the Hess–Murray law or ‘the principle of minimum work’.

## 1. Introduction

Fractals are everywhere [1]

Branched or dendritic structures abound in nature [1–9]. The design is not a fortuitous evolutionary outcome [8] but is pretty much an adaptive architecture fashioned by the universal laws of physics and tweaked by the pressures of natural selection [10–20]. From the remarkable similarity between the bronchial system of the human lung and that of an inverted botanical tree, the airway system of the human lung is commonly called the ‘respiratory tree’ [2,5,21]. Fractal geometry (FG) is a branch of mathematics that characterizes the structure of complex structures [2,5,22–24]. Utilizing FG based algorithms, Kitaoka & Suki [25] and Kitaoka *et al.* [26] prepared three-dimensional (3D) computational models that resembled the structure of the human lung. Historically, branched structures have aroused human curiosity for a long time. Later corroborated by (among others) Richter [27], Leonardo da Vinci (1452–1519) determined that within each generation, the cross-sectional area of a tree trunk is equal to the sum of the cross-sectional areas of the branches. The advancement of FG from applied mathematics to life sciences [28,29] transformed the hitherto speculative and in some cases teleological interpretation of form and function. The etymology of the word ‘fractal’ is from Latin *‘frāctus’*, which corresponds with the English words of ‘broken’, ‘fractured’ and ‘fraction’. A fractal dimension (*D*_F_) states the structural complexity of an assemblage [2,5,30,31]. Typically, it is a fraction or a non-integer number [2,5,32,33]. In the conventional Euclidean geometry, the topological dimensions are finite numbers (i.e. they are integers), with a point having 0 dimension, a line 1 dimension, a plane 2 dimensions and a cube, a sphere and a cylinder 3 dimensions. Whole numbers (i.e. integers) cannot sufficiently detail the design of a complex natural structure [1,2,5,34–37]. Michael [37] termed FG as ‘the geometry between dimensions'. It allows the non-topological properties of form and shape to be more well-captured [5]. While the so-called absolute or mathematical fractals are space-filling and self-similar over an infinite range of magnification [2], among others, Weibel [5], Captur *et al.* [24], Florio *et al.* [38], Avnir *et al.* [39] and Fernández *et al.* [40] have argued that biological structures are quasi-fractal structures (i.e. they are space-filling only to an extent and may display fractility only in some parts of their assembly or over a finite range of magnification). The complex branched architecture of structures such as the bronchial- and the vascular systems of the mammalian lung, the river drainage basins, the root systems of plants, the brain folds, the vascular systems of organs like the kidney and the neural networks of the brain is fractal [2,5,8,23,34,41–46]. Weibel [5] stated that ‘fracticality could explain life's design principles’, while Mandelbrot [2] espoused that ‘the lung can be self-similar and it is'. In complex multicellular organisms, life is sustained by an efficient networked infrastructure by which vital materials and substances such as nutrients and oxygen are delivered to all parts and information transmitted by electrical signals in the form of nerve impulses for coordination of physiological processes. Mostly developed about a century ago, the Hess–Murray law (H-ML) [10–12,47] is an elemental physical principle that expresses the relationship between the morphologies of the branched and the energetic cost of transporting fluids through tubular structures [48–57]. The H-ML has been mathematically and empirically substantiated by among others Cohn [13,14], Uylings [15] and LaBarbera [17]. It states that in natural transporting systems such as blood vessels, laminar flow occurs with minimum energy loss [17,49,51,58–60]. Considerably based on the founding paradigm of the H-ML, the more inclusive ‘constructal law of design and evolution’ was more recently posited [8,60–62].

The branched airways and blood vessels of the human lung have been quantitatively well investigated [42,63–70], but their *D*_F_ values have been determined only in few studies [30,71–76]. Replicas, images and models have been prepared using different materials and methods and measurements made [70,77–83]. The *D*_F_ values have been calculated using among other methods box counting [2,22,24,28,29,33,84–89], grey level co-occurrence matrix [90] and perimeter-to-area measurement [37]. Called the ‘new approach’, recently, a mathematical approach that was based on one of the variants of the Von Koch curve [91] was used to calculate the *D*_F_ of the human lung from data reported by other investigators. *D*_F_ values have also been determined by double logarithmic plots of the diameters and the lengths of the branches of structures such as the bronchial and vascular systems of the human lung [5,30,92,93]. Although the *D*_F_ values convey the same general detail (i.e. structural complexity), those values obtained by digitized computational methods are not exactly the same as those obtained from measurements of diameters and lengths of the branches. Designated as ‘the geometry of life’ [5] and ‘the fourth dimension of life’ [94], FG is a heuristic understanding of the basis of the designs of complex biological structures [5,30,73,74,95,96]. It has found important applications in different areas of medicine such as tissue and organ engineering [97], and quantitative differentiation of normal from diseased and pathological tissues [33,36,98–105]. Hughes [20] noted that disease is a consequence of change from optimal design; in fatal asthma cases, Boser *et al.* [106] observed a significant decrease of the *D*_F_ which they ascribed to a decrease in the extent of space-filling of the branches of the airways; Mauroy *et al.* [107] pointed out that during asthmatic attacks, bronchial malfunction stems from the unoptimized structure of the pulmonary bronchial tree; Liew *et al.* [108] and Gould *et al.* [109] reported that suboptimal space-filling architecture causes organs to perform poorly and the best performance of a space-filling structure emanates from a balance between under-exploitation and over-exploitation of the blood–gas barrier by the oxygen molecules; and King *et al.* [110] stated that in cases of Alzheimer's disease, *D*_F_ (which is a measure of the functional connectivity of the neurons in the brain) decreases as the condition progresses clinically. Also, fractility has been employed to identify and quantitatively diagnose conditions such as pulmonary hypertension [33,111–113]; heart rate has been noted to become more regular before heart attacks [114,115]; non-optimal branching geometry of a structure constitutes an undesirable risk factor during the early stages of life [20]; pathological conditions such as atherosclerosis and calcification derive from departure from optimality (i.e. non-compliance with the H-ML) [116–118]; and functional efficiency stems from the fractility [119]. Here, the diameters and the lengths of the branches of the different generations and the angles of bifurcation of the bronchial- and vascular systems (pulmonary artery and pulmonary vein) of the human lung were measured on latex rubber cast preparations and the *D*_F_ values determined by double logarithmic plots of the measurements.

## 2. Results

### 2.1. Morphologies of the bronchial and vascular systems

**Figure 1.**
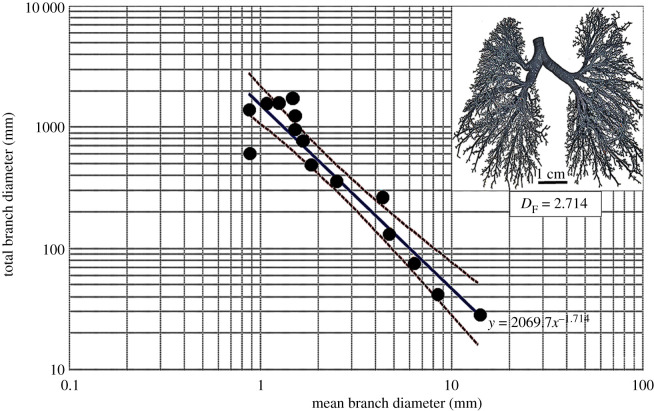
Double logarithmic plot of the total branch diameter against the mean branch diameter of the bronchial (airway) system of the cast human lung. The fractal dimension (*D*_F_) was 2.714. The insert shows the cleared cast of the bronchial system on which measurements were made. The dashed lines are the 95% confidence interval lines of the plotted data.

**Figure 2.**
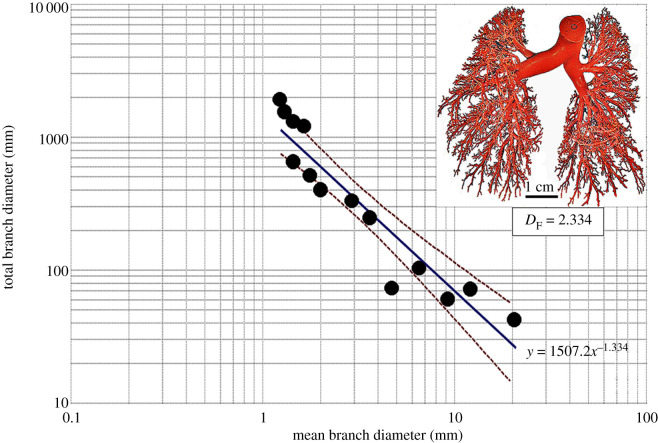
Double logarithmic plot of the total branch diameter against the mean branch diameter of the pulmonary vein of the human lung. The fractal dimension (*D*_F_) was 2.334. The insert shows the cleared cast of the pulmonary vein on which measurements were made. The dashed lines are the 95% confidence interval lines of the plotted data.

**Figure 3.**
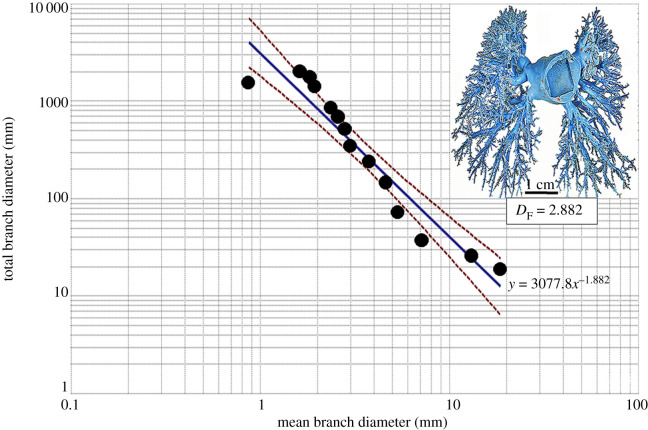
Double logarithmic plot of the total branch diameter against the mean branch diameter of the pulmonary artery of the human lung. The fractal dimension (*D*_F_) was 2.882. The insert shows the cleared cast of the pulmonary artery on which measurements were made. The dashed lines are the 95% confidence interval lines of the plotted data.

The casts of the bronchial (airway) and vascular (pulmonary artery and pulmonary vein) systems displayed dichotomous asymmetrical branching with irregular branch diametric and length sizes and angles of bifurcation (figures 1–3). While most of the angles of bifurcation were oriented perpendicular to the direction of gravity in an erect (normal) standing position, a few of them were inclined to the perpendicular direction at various angles.

**Figure 4.**
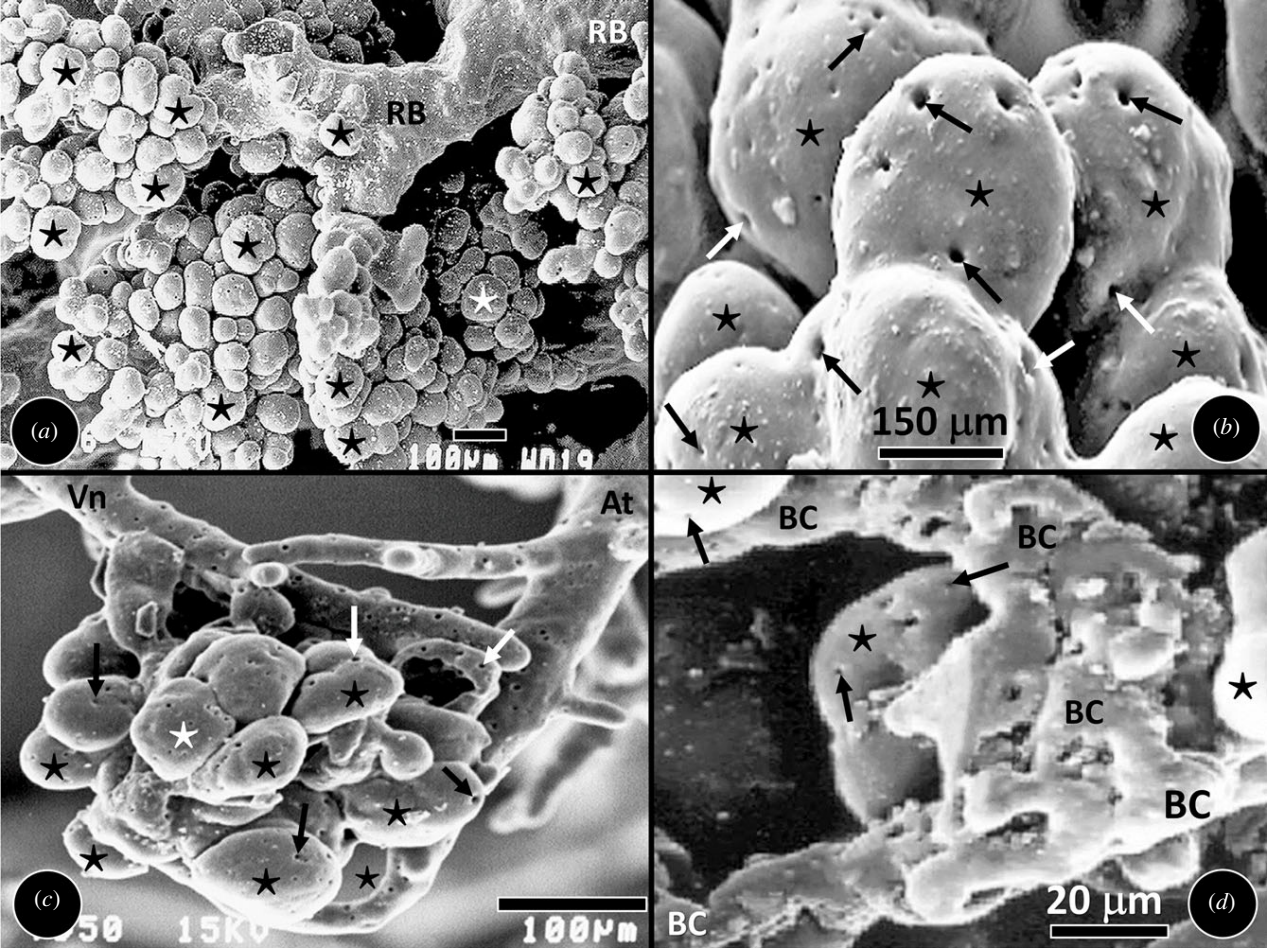
Scanning electron micrographs of the terminal parts of the casts of the bronchial and vascular systems of the human lung. (*a*) The normal morphologies of the alveoli (stars) and blood capillaries shows that the casting material was suitable and casting method was proper. RB, respiratory bronchioles. (*b*) The respiratory bronchioles seen giving rise to alveoli (stars) which are interconnected by the interalveolar pores or the eponymous pores of Kohn (arrows). (*c*) A cluster of alveoli (stars) that are supplied with blood by an arteriole (At) and drained by a venule (Vn). Arrows, interalveolar pores. (*d*) Interfacing between the alveoli (stars) and the blood capillaries (BC) at the gas exchange level. Arrows, interalveolar pores.

The casts of the bronchial system, the pulmonary artery and the pulmonary vein presented normal morphological features of the human lung (figure 4*a–d*). The terminal components of the airway (i.e. the alveoli) and the vascular systems (i.e. the blood capillaries) displayed normal shapes and sizes. The interface between alveoli and the blood capillaries, where gas exchange occurs, was clearly observed (figure 4*d*).

### 2.2. Measurements of the bronchial and vascular systems

The branches of the three main parts of the lung (i.e. the bronchial, the pulmonary artery and the pulmonary vein systems) were categorized using the ‘morphogenetic’ or ‘regular dichotomy’ or ‘Weibel's’ ordering method [1,5,40,41,95]. The mean diameters and the mean lengths of the branches that comprise the different generations of the main systems and the angles of bifurcation of the branches are shown in tables 1–3; the values of *D*_F_ which were determined here and those that have been reported by other investigators on the human lung are shown in table 4; and comparison of the *D*_F_ values of the lungs of the non-human vertebrates and other natural structures that have been investigated are given on table 5. For the bronchial system, the pulmonary artery and the pulmonary vein, respectively, 15, 14 and 14 generations that comprised 8663, 7531 and 7355 branches were measured. The mean diameters of the branches (mm) of these systems were respectively 3.48 ± 3.54, 3.66 ± 3.11 and 4.46 ± 5.38. The coefficients of variation (CV) (%) of the mean diameter and length measurements for the bronchial, pulmonary artery and pulmonary vein systems were respectively 29.78 ± 11.47 and 28.40 ± 13.76, 28.25 ± 7.15 and 35.23 ± 13.30, and 103.81 ± 73.82 and 40.85 ± 13.37; the mean ratio of the change in the mean generation diameters and the mean generation lengths were respectively 1.24 ± 0.23 and 0.86 ± 1.30, 1.13 ± 0.43 and 1.16 ± 0.35, and 1.28 ± 0.27 and 1.12 ± 0.22. Regarding the mean length-to-diameter ratio of the branches that formed the generations, for the bronchial system, the pulmonary artery and the pulmonary vein, the values were respectively 3.86 ± 1.27, 3.82 ± 1.71 and 3.55 ± 1.29, while the mean ratios of the change in the number of branches with the generations were respectively 1.55 ± 0.72, 1.68 ± 0.67 and 1.66 ± 0.68. The total diameters (mm), which were calculated by multiplying the number of branches of a generation with the mean diameter for that generation, were respectively 750.40 ± 598.79, 743.22 ± 657.23 and 744.73 ± 683.37 for the bronchial system, the pulmonary artery and the pulmonary vein. Respectively, for the bronchial system, the pulmonary artery and the pulmonary vein, the mean bifurcation angles (in degrees) were 34.23 ± 6.20, 33.83 ± 6.03 and 32.06 ± 1.69, while the mean ratios of the change in the mean angles of bifurcation were 1.04 ± 0.18, 1.05 ± 0.23 and 1.05 ± 0.18, values which were very close to each other.

**Table 1.** Morphometric parameters of the generations of the bronchial system of the human lung. s.d., standard deviation.

generation number	number of branches	ratios of change in the number of branches	mean diameters of the branches (mm) s.d.	mean coefficient of variations of the diameter measurements (%)	ratios of change in the mean diameters of the branches	total diameters of the branches (mm)	mean lengths of the branches (mm) ± s.d.	mean coefficient of variations of the length measurements (%)	ratios of change in the mean lengths of branches	length–diameter ratios of the branches	mean angle of bifurcation in degrees	ratios of change in mean angles (in degrees) of bifurcation
1	2	2.5	14.02 ± 1.73	12.34	—	28.04	36.50 ± 5.12	14.03	—	2.60	43.1	—
2	5	2.4	8.34 ± 1.27	15.23	1.68	41.70	11.23 ± 4.81	42.83	3.25	1.35	52.2	0.83
3	12	2.3	6.32 ± 3.31	52.37	1.32	75.84	11.98 ± 3.37	28.13	0.94	1.90	35.7	1.46
4	28	2.21	4.74 ± 0.89	18.78	1.33	132.72	11.37 ± 3.56	31.31	1.05	2.40	32.9	1.09
5	62	2.37	4.27 ± 0.83	19.44	1.11	264.74	11.36 ± 4.37	38.47	1.00	2.66	28.8	1.14
6	147	1.84	2.46 ± 0.62	25.20	1.74	361.62	10.46 ± 2.83	27.06	1.09	4.25	29.4	0.98
7	270	1.67	1.83 ± 0.76	41.53	1.34	494.10	7.760 ± 3.11	40.08	1.35	4.24	33.2	0.89
8	452	1.40	1.69 ± 0.67	39.65	1.08	763.88	8.400 ± 4.85	57.74	0.92	4.97	28.5	1.16
9	632	1.30	1.52 ± 0.71	46.72	1.11	960.64	8.260 ± 3.65	44.18	1.02	5.43	26.7	1.07
10	821	1.45	1.49 ± 0.57	38.26	1.02	1223.29	7.720 ± 2.44	31.61	1.07	5.18	36.7	0.72
11	1193	1.10	1.46 ± 0.41	28.08	1.02	1741.78	6.190 ± 2.22	35.86	1.25	4.24	33.4	1.09
12	1312	1.10	1.22 ± 0.32	26.23	1.20	1600.64	5.930 ± 1.68	28.33	1.04	4.86	34.8	0.96
13	1443	1.10	1.09 ± 0.28	25.69	1.12	1572.87	5.470 ± 0.77	14.08	1.08	5.02	30.6	1.14
14	1586	0.44	0.87 ± 0.30	34.48	1.25	1379.82	3.910 ± 0.48	12.28	1.40	4.49	35.7	0.86
15	698	0.00	0.88 ± 0.20	22.73	0.99	614.24	3.790 ± 0.38	10.03	1.03	4.31	31.8	1.12
mean/total	8663	1.55 ± 0.72	3.48 ± 3.54	29.78 ± 11.47	1.24 ± 0.23	750.40 ± 598.79	10.02 ± 7.55	28.40 ± 13.76	1.25 ± 0.59	3.86 ± 1.27	34.23 ± 6.20	1.04 ± 0.18

**Table 2.** Morphometric parameters of the generations of the pulmonary artery of the human lung. s.d., standard deviation.

generation	number of branches	ratios of change in the number of branches	mean diameters of the branches (mm) ± s.d.	mean coefficient of variations of the diameter measurements (%)	ratios of change in the mean diameters	total diameters of the branches (mm)	mean length of the branches (mm) ± s.d.	mean coefficient of variations of the length measurements (%)	ratios of change in the mean lengths of the branches	length–diameter ratios of the branches	mean angle of bifurcation in degrees	ratios of change in the mean angles (in degrees) of bifurcation
1	2	2.5	12.94 ± 2.51	19.40	—	25.88	31.50 ± 2.26	7.17	—	2.43	52.7	—
2	5	2.8	7.32 ± 1.87	25.55	1.76	36.60	14.67 ± 3.88	26.45	2.16	2.00	32.5	1.62
3	14	2.29	5.23 ± 1.27	24.28	1.05	73.22	13.07 ± 4.06	31.06	0.85	2.50	34.2	0.95
4	32	1.97	4.68 ± 1.73	37.00	0.66	149.76	11.36 ± 3.35	29.49	1.15	2.43	31.2	1.10
5	63	1.83	3.73 ± 1.26	33.78	1.10	234.99	10.14 ± 4.36	43.00	1.12	2.72	30.8	1.01
6	115	1.70	2.96 ± 0.98	33.11	1.02	340.40	12.12 ± 3.41	28.14	0.84	4.09	33.1	0.93
7	196	1.47	2.76 ± 0.67	24.28	1.36	540.96	8.38 ± 3.60	42.96	1.45	3.04	34.3	0.97
8	289	1.27	2.47 ± 0.63	25.51	0.95	713.83	8.59 ± 5.31	61.82	0.98	3.48	35.5	0.97
9	367	1.97	2.30 ± 0.59	25.65	0.99	844.10	8.58 ± 4.96	57.81	1.00	3.73	27.7	1.28
10	724	1.28	1.93 ± 0.71	36.79	0.70	1397.32	8.82 ± 2.96	33.56	0.97	4.57	26.2	1.06
11	927	1.37	1.83 ± 0.50	27.32	1.34	1696.41	6.43 ± 2.43	37.79	1.37	3.51	37.3	0.70
12	1274	1.38	1.64 ± 0.45	27.44	1.00	2089.36	6.26 ± 2.44	38.98	1.03	3.82	30.1	1.24
13	1753	0.00	0.87 ± 0.41	47.13	0.58	1525.11	5.97 ± 1.88	31.49	1.05	6.86	31.9	0.94
14	1770	—	0.63 ± 0.14	22.22	2.12	737.10	5.23 ± 1.23	23.52	1.14	8.30	36.1	0.88
mean/total	7531	1.68 ± 0.67	3.66 ± 3.11	28.25 ± 7.15	1.13 ± 0.43	743.22 ± 657.23	10.79 ± 6.35	35.23 ± 13.30	1.16 ± 0.35	3.82 ± 1.71	33.83 ± 6.03	1.05 ± 0.23

**Table 3.** Morphometric parameters of the generations of the pulmonary vein of the human lung. s.d., standard deviation.

generation	number of branches	ratios of change in the number of branches	mean diameters of the branches (mm) ± s.d.	mean coefficient of variations of the diameter measurements (%)	ratios of change in the diameters of the branches	total diameters of the branches (mm)	mean lengths of the branches (mm) ± s.d.	mean coefficient of variations of the length measurements (%)	ratios of change in the mean lengths of the branches	length–diameter ratios of the branches	mean angles in degrees	ratios of the change in the mean angles (in degrees) of bifurcation
1	2	3.00	20.97 ± 1.66	7.92	—	41.94	16.75 ± 3.27	19.52	—	0.80	46.4	—
2	6	2.67	11.81 ± 3.23	27.35	1.78	70.86	18.88 ± 3.86	20.45	0.89	1.60	31.2	1.49
3	16	2.25	6.55 ± 2.14	32.67	1.80	104.80	15.80 ± 4.15	26.27	1.19	2.41	34.3	0.91
4	36	1.89	4.66 ± 1.53	32.83	1.41	74.56	10.64 ± 4.41	41.45	1.49	2.28	28.5	1.20
5	68	1.72	3.65 ± 2.67	73.15	1.28	248.20	9.18 ± 4.23	46.08	1.16	2.52	29.5	1.12
6	117	1.69	2.85 ± 3.78	132.63	1.28	333.45	11.73 ± 3.62	30.86	0.78	4.12	32.4	0.91
7	198	1.47	1.98 ± 4.56	230.30	1.43	392.04	8.14 ± 4.35	53.44	1.44	4.11	34.1	0.95
8	292	1.53	1.74 ± 2.45	140.80	1.14	508.08	7.82 ± 4.31	55.12	1.04	4.49	26.3	1.30
9	447	1.69	1.45 ± 1.85	127.59	1.20	648.15	6.23 ± 3.94	63.24	1.26	4.30	27.9	0.94
10	754	1.21	1.63 ± 1.67	102.45	0.89	1229.02	8.13 ± 3.48	42.80	0.77	5.00	30.4	0.92
11	912	1.30	1.47 ± 2.05	139.46	1.11	1340.64	6.27 ± 2.47	39.39	1.30	4.27	30.2	1.01
12	1186	1.30	1.29 ± 3.46	268.22	1.13	1529.94	6.16 ± 3.42	55.52	1.02	4.78	30.3	1.00
13	1542	0.00	1.24 ± 1.09	87.90	1.04	1912.08	5.50 ± 1.97	35.82	1.12	4.44	31.6	0.96
14	1779	—	1.12 ± 0.56	50.00	1.11	1992.48	5.13 ± 2.15	41.91	1.07	4.58	35.7	0.89
mean/total	7355	1.66 ± 0.68	4.46 ± 5.38	103.81 ± 73.82	1.28 ± 0.27	744.73 ± 683.37	9.75 ± 4.30	40.85 ± 13.37	1.12 ± 0.22	3.55 ± 1.29	32.06 ± 1.69	1.05 ± 0.18

**Table 4.** Fractal dimensions (*D*_F_) of the bronchial and the vascular systems of the human lung reported by different investigators using different methods.

investigators	this study		values determined by the ‘new approach model’ of Lamrini-Uahabi & Atounti [91] on data obtained in this study	Boser *et al.* [106]	Kitaoka & Takahashi [120]	Lamrini- Uahabi & Atounti [91]	Weibel [5]	Varner & Nelson [75]	Huang & Yen [96]	Nelson & Manchester [30]		Nelson & Manchester [30]	Nelson & Manchester [30] reanalysis of data by Weibel [63] and Weibel and Gomez [64]^٭^
method	latex casting and mathematical modelling			latex casting and box counting of pixels of grey-scaled photographic images	digital images of the reconstructed airways	mathematical modelling using the so-called ‘new approach’	mathematical modelling	computational modelling of data generated by box counting method	latex casting and mathematical modelling	reanalysis of data published by Horsfield & Cumming [121]		reanalysis of data published by Raabe *et al.* [65]	review of published data
*D*_F_ : airways	2.714^a^	3.098^b^	2.836^a^	1.84^c^	1.74^a^	2.88**^b^**	2.35^a^	2.0^c^	—	—	2.64^a^	2.76^b^	4.10^b^
*D*_F_ : veins	2.334^a^	4.041^b^	2.728^a^	—	—	—	2.64^a^	—	2.64^a^	2.86^b^		—	—
*D*_F_ : arteries	2.882^a^	3.916^b^	2.678^a^	—	—	—	2.71^a^	—	2.71^a^	2.97^b^		—	—

^a^*D*_F_ values calculated using diameter measurements of the generations.

^b^*D*_F_ values calculated using length measurements of the generations.

^c^*D*_F_ values determined by computational methods.

**Table 5.** Fractal dimensions (*D*_F_) of different biological structures and non-human lung.

investigators	Calkins [119]	Bhandari *et al*. [102]	Pantic *et al*. [90]	Gan *et al*. [92]	Karperien & Jelinek [89]	Frisch *et al*. [122]	Liang *et al*. [88]	Youlin & Lede [123]
method	box Counting of computerized scans	box Counting of scanning electron micrographs	box counting of micrographs	latex casting and mathematical modelling	computerized modelling	box counting of grey-scaled micrographs	box counting and mathematical modelling	mathematical modelling
structural tissue/system studied	human retina	Stage 1 colon cancer	human kidney medulla	dog pulmonary venous system	human microglial cells	rat medial collateral ligament	grass roots	jungle river basin
*D*_F_	1.617	1.882	1.8494	2.99	1.58	1.807	2.437	1.75

### 2.3. *D*_F_ values of the bronchial system, the pulmonary artery and the pulmonary vein

From the measurements of the diameters and the lengths of the branches, respectively, the *D*_F_ values of the bronchial system, the pulmonary artery and the pulmonary vein were 2.714 and 3.098, 2.882 and 3.916, and 2.334 and 4.041.

This is the first study where the three main systems of the human lung have been cast together, analysed and the data modelled to determine *D*_F_. Since the lung largely comprises air, blood and compliant tissue, casting of single systems, as has been done by some investigators [73,92], is accompanied by certain technical difficulties that include possible over-distension of the branches during casting, a process that is constrained during simultaneous casting. Although it has yet to be proven, the replicas prepared here may turn out to be some of the most representative that have been investigated in comparison with similar studies. Furthermore, the quality of the casts may have been greatly improved by the fact that despite the many necessary stages that have to be followed after death before a human body is released for dissection and/or research, here, conscious effort was made to acquire the cadaver in as short a time as possible, and the whole time it was kept and cast in a cold room. This should have minimized autolytic changes of the pulmonary tissues.

## 3. Discussion

Casting with various materials has been and continues to be a meaningful technique of studying biological structures [70,78–80,124,125]. Latex rubber was used here because of the following reasons: (i) it is nontoxic and is thus safe to handle; (ii) it is water-soluble and therefore its viscosity can be easily varied to suit the organ cast; (iii) it can be easily coloured differently for parts of the structure to be cast and easily differentiated; (iv) depending on the level of dilution, it sets rapidly and hence results can acquired faster; and (v) it shrinks little, if at all, and consequently few, if any, distortions form [70,79,80]. The casts of the bronchial system, the pulmonary artery and the pulmonary vein which were prepared here displayed the normal morphologies of the human lung [63,64,75,95,106,119,124] (figure 4*a–d*) which corresponded with those reported by other investigators [42,43,95,124,126]. It showed that the casting material used and the method applied for casting was appropriate.

**Figure 5.**
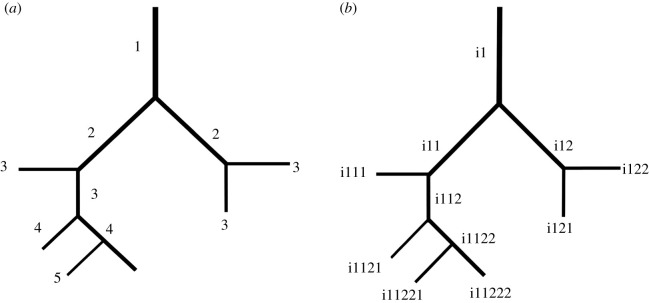
(*a*) The morphogenetic (Weibel's) ordering method used to categorize the branches of the bronchial and vascular systems of the cast of the human lung. The branches were systematically ordered from the trachea outwards. (*b*) To ensure that branches were not measured twice, a binary system was adopted to order the branches. The codes (i1) were based on those allocated to the parent branches, with the daughter branches being labelled in numerical order from left to right.

**Figure 6.**
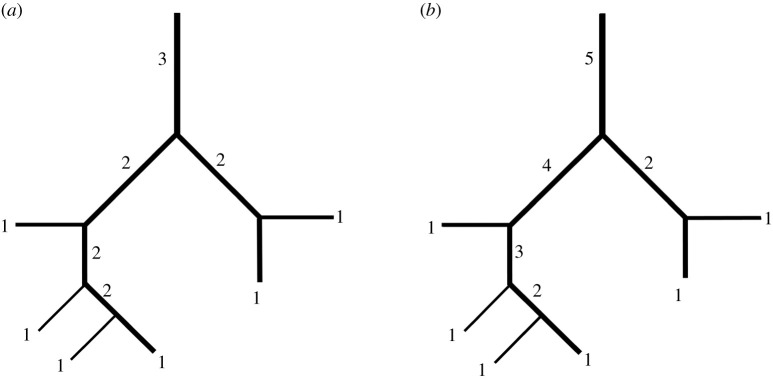
(*a*) Strahler's and (*b*) Horsfield's ordering methods. In both cases, the ordering starts from the periphery and advances inwards (i.e. towards the trachea). For Strahler's method, where two branches of the same order meet, the parent branch increases in order by 1 while if two branches belonging to different orders meet, the ensuing branch is allocated the order of the highest ordered branch of the pair. In Horsfield's ordering method, the lowest order is allocated to the smallest branch and the order of a parent branch depends on the orders of its daughter branches: the parent branch is given an order value that is one higher than the highest order assigned to one of its daughter branches.

Structurally, the human lung comprises three main parts, namely the bronchial system, the pulmonary artery and the pulmonary vein. Topographically, the bronchial system and the pulmonary artery closely track each other while the pulmonary vein and its branches occupy an intermediate position between the broncho-arterial units [95]. By any criterion, the human lung is a structurally complex organ [2,42,43,63,64,95,107]. The fractal properties of its parts have been investigated to understand its structure and function in health and disease states [2,5,89,95,101,127]. Various *D*_F_ values have been determined for the bronchial and the vascular systems of the human lung [5,30,88,89,91,92,106] (table 4). The morphogenic (Weibel's) ordering method [2,30,65,95,126,128,129] (figure 5*a*,*b*) has been used to categorize the branches that form the various generations of the lung [63,64], while the ‘older’ ordering method of Strahler [73,130–132] (figure 6*a*), which was initially developed to study geomorphological (landscape) features such as river drainage systems, has also been applied on some biological structures. Modifications of Strahler's ordering method such as Horsfield's ordering method [133] (figure 6*b*) and the diameter-defined Strahler's ordering method [67,73,92,134,135] were developed to improve the erstwhile (Strahler's) model. For the morphogenetic ordering method, the branches are classified according to the succession they formed during the development of the organ [63–65,95,126,129]. The model assumes that regular dichotomy and that the branches are equal in size [63,64,95]. By considering the irregularity of the bifurcation pattern, Strahler's ordering method may reduce the variation of the measurements made on the branches [95,130–132]. The ordering starts from the periphery and advances inwards (i.e. towards the trachea; figure 6*a*). When two branches of identical order meet, the convergent branch number increases by one, while if two branches belonging to different orders meet, the resulting branch is allocated the order of the highest-ordered branch of the pair [31,73,92]. In Horsfield's ordering method [68,69,136–138] (figure 6*b*), the lowest order is assigned to the smallest branch (i.e. the ordering of a parent branch depends on the orders of its daughter branches and the parent branch is given an order value that is one higher than the highest order assigned to one of its daughter branches). With the exception of a condition where the symmetrical hierarchical arrangement of branches exists [129], generally, Strahler's ordering method yields fewer generations compared with Horsfield's [95,138]. Although the application of Strahler's [130–132] and Horsfield's ordering methods [68,69,138,139] could have reduced the variability between the measurements which were made on the branches in this study, for the reasons given below, the morphogenetic ordering method was preferred. We subscribed to the consideration of Weibel [95] and Hsia *et al.* [129] that for the human lung, the morphogenetic ordering method provides more instructive data for understanding physiological processes such as flow dynamics [95,138,140,141] and particle deposition [142,143], while Strahler's ordering method yields more meaningful data for the pathologists [129,138]. Furthermore, Horsfield [138] cautioned that a great deal of information is lost in the simplification inherent in Strahler's ordering method, especially with regard to the connectivity of the branches. It is important to note that the two main ordering models (Weibel's and Strahler's) are not at total variance. Weibel [95] remarked that although the morphogenetic and Strahler's ordering methods are ‘conceptually different’, ‘both approaches lead to the same conclusions', while Horsfield [138] observed that the morphogenetic and Strahler's ordering methods ‘are not in conflict with each other but are simply looking at different aspects of the same thing’. Together with the considerations above, the morphogenetic ordering method was applied in this study for the following reasons: (i) the primary aim of this study was to acquire data which informed the structure and function of the human lung; (ii) the model emulates the development of the airway- and the vascular systems of the lung and may, therefore, yield most explicatory data [144,145]; and (iii) nature's accommodation of the pressures of natural selection generates the best possible solutions to the challenges of life [8,48,60,146–152]. Molecular biology studies have shown that the vertebrate lung develops by a well-orchestrated spatio-temporal expression of an assortment of morphogenetic cues (molecular factors) which by an iterative process assembles a branched design [153–160].

Complex biological entities possess various *D*_F_ values [106] which may be determined by factors such as the stage of development, lifestyle and whether the structure is healthy or diseased [161]. Chau [162] advised that in order to absolutely capture the fractility of a structure, different analytical techniques should be used to obtain data at various magnifications; Captur *et al.* [24], Avnir *et al.* [39] and Boser *et al.* [106] contended that branched structures do not display self-similarity over infinite scales of magnification as argued by Mandelbrot [2] but are only space-filling structures; and Nelson & Manchester [30], Falconer [35] and Lennon *et al.* [36] stated that although the structural systems of the lung may not display self-similarity over an infinite range of magnification and only some parts may be fractal [30,35,36]. Regarding the human lung, different *D*_F_ values have been reported by various investigators on the bronchial and vascular systems [5,30,64,73,75,91,106,120] (table 4). For the bronchial system, which has been investigated to a greater extent compared with the vascular system, the *D*_F_ values range from 1.75 to 3.098. Also, differences exist between the *D*_F_ values of the human lung and those of the lungs of non-human vertebrates and other branched natural structures [88–90,92,102,119,122,123] (table 5). Morphological differences, variations of the methods of the preparation of the formations on which measurements are made, the ordering method employed to classify the branches and the mathematical models used to determine/calculate *D*_F_ may account for the variations. Here, the diametric measurements gave *D*_F_ values of 2.714 for the bronchial system and 2.882 for the pulmonary artery (table 4), values particularly close that of 3, which is expected for a space-filling tree-like structure [1,2,5,95]. The *D*_F_ determined in this study for the bronchial system (2.714) was very close the value of 2.760 reported by Nelson & Manchester [30] from measurements of the lengths of the branches of the airways of the human lung, which were categorized by the morphogenetic ordering method [65]. Nelson & Manchester [30] dismissed a *D*_F_ of 4.10, which they calculated from data reported by Weibel [63] and Weibel & Gomez [64] on the human lung, as having no ‘physical significance’. Where length and diameter measurements have been used to determine *D*_F_ values by double logarithmic plotting, regarding the dog's pulmonary venous system, Gan *et al.* [92] reported a higher *D*_F_ (2.99) from lengths and a smaller one (2.489) from diameters. Here, lengths gave higher *D*_F_ values compared with diameters (table 4). In a rigid tube, during laminar flow, the diameter has a greater effect on the flow dynamics compared to length [163–165]. In accordance with the Hagen–Poiseuille law of fluid flow, which is expressed as Δ*P* = 8*l* µV/μr^4^ [165] (where Δ*P* is the pressure difference between the ends of the tube; *l* is the length of the tube; *μ* is the fluid dynamic viscosity; *V* is the volumetric flow rate; and *r* is the radius of the tube), resistance is inversely proportional to the radius and directly proportional to length. Decreasing the radius of the tube by one-half increases resistance 16-fold (i.e. by a factor of 2^4^), while doubling length increases resistance two-fold. The closeness of the values of *D*_F_ that were determined in this study from diameter measurements, especially for the bronchial system and the pulmonary artery, to the expected value of 3 of an absolutely space-filling structure [1,2,28,29] may, to an extent, be ascribed to the great significance of diameter as a structural parameter in determining fluid flow. For the human lung, Huang *et al.* [73] noted that the diameters of the branches of the pulmonary artery and vein were constant, while Phillips & Kaye [166] observed that to a greater extent air flow in the lung is determined by the diameters of the airways, a feature well-evidenced during asthmatic attacks [163]. Consistency of the diameters and lengths of the branches was observed in this study: for the bronchial system, the pulmonary artery and the pulmonary vein, the mean diametric and length changes were respectively 1.24 and 1.25, 1.13 and 1.16, and 1.28 and 1.12, values which were both close to each other and close to the value of approximately 1 reported by Phillips & Kaye [166] on the bronchial system of the human lung and said to display optimal air flow.

Optimization is quantitatively defined as maximization of output or performance for a certain input or cost [48,148,150,151,167]. It is an adaptive process that occurs in accord with the universal laws of physics and is tweaked by the pressures of natural selection [2,5,76,94,95,146–152,168]. The ‘principle of minimum work’ or the H-ML [10–12,47,169] is one such law. The subject matter has been reviewed by among others LaBarbera [17,58], Hughes [20], Sherman [49], Sciubba [56], LaBarbera & Vogel [170] and Xu *et al.* [171]. While it has been challenged by some investigators [149,172], the branching angles of fluid transporting structures are an important structural feature that permits compliance with the H-ML [12,15,173–177]. Originally developed for the specific case of the cardiovascular system in which blood is transported through a single branching tube [11,12,47,152], the H-ML defines the cost of laminar flow through passageways. In the animal kingdom, structures that obeyed the H-ML are reported to have developed as long ago as approximately 375 Ma and may have since evolved independently at least three times [51,59]. In dendritic transporting structures, optimality exists where the cube of the parent (i.e. upstream) channel radius is equal to the sum of the cubes of the daughter (i.e. downstream) conduits' radii. Mathematically, the relationship is expressed as follows: $r_{0}^{x}=r_{1}^{x}+r_{2}^{x}+\ldots \;r_{z}^{x}$, where the subscripts denote the parent (0) and the daughter branches (1, 2, …, *z*) and the superscripts (*x*) are the junctional- or branching exponents [47,56,130–132]. For certain vascular morphologies, Takahashi [31] determined that the *D*_F_ values and the branching exponents (*x*) were equal (see Hughes [20] for succinct re-verification of the relation). The H-ML is obeyed in many branched biological structures [8,15–17,20,49,53,58,121,130–132,174,178–185]. The data acquired here compellingly show that the bronchial and vascular systems of the human lung comply with the H-ML. The branching ratio of the bronchial system of the human lung (i.e. the total number of branches in one order to that in the next one), which was reported by Weibel [95] to indicate optimal structure, was approximately 1.4, a value close to that of 1.55 for the same system found in this study. The mean branching angles of the bronchial system (34.23°), the pulmonary artery (33.83°) and the pulmonary vein (32.06°) obtained here fell within the range of the values of 27–40° for blood vessels and airways expressed to be optimal angles of bifurcation by several investigators [11,15,56,140,186,187]. Regarding the carotid artery, which had a branching angle ratio of 1.2, blood flow was reported to obey the H-ML [140]. Here, the mean ratios of the angles bifurcation for the bronchial, pulmonary artery and pulmonary vein systems were respectively 1.04, 1.05 and 1.05, values that are close to each other and also to the value (1.2) for the carotid artery [140]. Showing morphological similarities, the mean ratios of the change of the number of branches of the bronchial system, the pulmonary artery and the pulmonary vein, which were respectively 1.55 ± 0.72, 1.68 ± 0.67 and 1.66 ± 0.68, were not statistically significantly different (*p* > 0.5). For optimal blood flow in branched blood vessels, Lorthois & Cassot [188] reported that the *D*_F_ values should range between approximately 2 and 3. Mandelbrot [2] determined that the diameter exponents of a space-filling tree-like structure was 3 and Weibel [95] showed that the diameters of the passageways decrease by the cube root of the branching ratio 2 (2^−1/3^ = 1.26), a feature which in terms of hydrodynamics instructs optimal flow. The internal carotid artery, which had a *D*_F_ of 2.9, complied with the H-ML [140]. Here, the *D*_F_ values of the bronchial system, the pulmonary artery and pulmonary vein, which were respectively 2.71, 2.88 and 2.334, were within the range of approximately 2–3 for the branched structures, which are reported to present optimal flow [2]. The *D*_F_ values of the bronchial system and the pulmonary artery that were determined here were close to the value of the carotid artery of 2.9, which obeyed the H-ML [140], and also close to the value of 3 of a space-filling structure [2]. In two human brains where the branching exponents ranged from 2.67 to 2.79 for arteries having diameters of less than 0.1 mm, blood flow complied with the H-ML [189]. After plotting the branch measurements of the bronchial system of the human lung against the generations on double logarithmic axes, Nelson *et al.* [71] and West *et al.* [76] found that lengths regressed with a slope of approximately −1.4 and diameters with that of −1.26, while Weibel [5] determined a slope of −1.35 for measurements of the same structure. Here the diameters of the bronchial system, the pulmonary artery and pulmonary vein respectively regressed by slopes of −1.71, −1.88 and −1.33. In the human lung, optimization of air flow occurs when the average length-to-diameter ratio of the branches is 3.25, and the diameters of the branches decrease by a factor of −0.86 and for the length by that of −0.62 [5]. In the open circulation of the blue crab (*Callinectes sapidus*), where the H-ML was reported to be obeyed, the mean segment (branch) length-to-diameter ratio was 3.98 [51]. Here, the corresponding values for the bronchial, pulmonary artery and pulmonary vein systems were respectively 3.86, 3.82 and 3.55, values close to that of 3.98 reported on the circulatory system of the blue crab [51]. The mean ratios of the decrease in the diameters and the lengths of the branches of the bronchial system, the pulmonary artery and the pulmonary vein that were determined here, which were respectively 1.24 and 1.25, 1.13 and 1.16, and 1.28 and 1.12, were close to the generation diameter decrease ratio of 1.26 (2^⅓^) of the bronchial system of the human lung, which has been reported to provide optimal air flow [5,63,64,95]. A branched structure, like the carotid artery, with a diameter decrease ratio of 1.26, obeys the H-ML [2,5,41,148]. In biological structures, there is lack of unanimity on what constitutes optimization [150,151,165,171,172,190,191] and whether the state/condition is achievable or even desirable [121,173–177]. Regarding the H-ML, some of the views of concern that have been expressed are the following: ‘perhaps Murray's law should be viewed as more of what you would call “guidelines” than actual rules' [20]; ‘optimum models are abstractions of biological systems and they are not expected to fit these systems with absolute accuracy’ [140]; and ‘there is a large spread between different parts of the circulation and possibly between different subjects in regard to the principal of minimum work’ [141]. In complete departure from the orthodox thinking that optimization is an adaptive (i.e. beneficial or favourable) state for the bronchial system of the human lung, Mouroy *et al.* [107] reported that it may not be desirable and may even be ‘dangerous’! To maintain the integrities of biological structures, optimization compels existence of safety factors [146,178,179,192] because the process renders the assemblages more susceptible to the stochastic events of nature. Complex trade-offs and compromises may be involved in the process of optimization [146,159,192]; transactions may not necessarily result in optimal outcomes.

While the CV of the means of the diameter and length measurements of the branches that comprised the generations of the pulmonary artery and bronchial systems, which were respectively 28.25% and 35.23%, and 29.78% and 28.40% (tables 1 and 2), were within a statistically acceptable range [193,194], the much greater CVs for the pulmonary vein (103.81 and 40.85%) (table 3) warrant comment. The greater heterogeneity of the sizes of the branches of the pulmonary vein may explain the higher CVs of the measurements. Aspects such as the ordering of the branches and the taking of the measurements would not be a factor because the procedures applied were the same for the three main parts of the lung. For the pulmonary vein, the mean diameter ratio change of the branches of 1.28 ± 0.27 was significantly greater (0.01 > *p* > 0.05) than that of the pulmonary artery (1.13 ± 0.43). For branched structures, Nelson & Manchester [30] observed that ‘the heterogeneity in branch size and number has led to several ordering schemes that give slightly different results'. Another property that may be thought to affect the measurements made on the pulmonary vein is that the blood vessel could be more compliant, a property which could cause enlargement of the branches with the application of casting pressure. While this might be the case for the systematic circulation, this is unlikely to occur in the pulmonary circulation, which is a low-pressure, high-flow system [5,95,195,196]. The pressure of the pulmonary artery that receives the entire output of the right heart is astonishingly only 15 mmHg (approx. 2 kPa) [95,194,195] compared with that of approximately 100 mmHg (13.33 kPa) in the systematic circulation [5,196]. Furthermore, from possible recruitment of blood capillaries that take up the increased vascular load, pulmonary vascular resistance drops when arterial or venous pressure increases [95,193]. The low pressure in the pulmonary circuit explains why the thicknesses of the walls of the branches of the pulmonary artery and vein are relatively much thinner compared with those of the blood vessels of the systemic circulation of similar luminal diameters [5,95,196]. Essentially, on histological sections, the pulmonary arteries and veins cannot be differentiated from the thicknesses of their walls [197–199]. There are no structural and functional differences between the pulmonary artery and vein that could cause variations in their compliance. Here, it was also found that for the pulmonary vein, variations in the diameters and the lengths of the branches along individual (single) paths were not significant.

In conclusion, to study the FG of branched structures, the branch-ordering method used should be rationalized. There is, however, some comfort in that although the methods may conceptually differ, they yield similar results. For studying the FG of the branched biological structures and especially the determination of their *D*_F_ values, various methods have been and continue to be used for preparation, analysis and modelling the data. These differences may in part explain the disparities in the published data. A model that integrates most, if not all, of the relevant structural parameters of branched structures is currently lacking. For example, the double logarithmic plot model of the diameters and lengths that was applied here entirely omits the bifurcation angles that are important structural properties associated with contributing to optimal flow across passageways. A simplified model may not adequately capture the complexity of a branched structure to give instructive *D*_F_ values. We echo the view expressed by Lamrini-Uahabi & Atounti [91] that ‘it would be ideal and very wise to find a unified value of the fractal dimension of lungs’. For the human lung, such a value would be of great importance in the diagnosis and treatment of pulmonary diseases and conditions such as asthma, emphysema, respiratory failure, pulmonary hypertension and pneumonectomy, and evaluation of pulmonary function in sports medicine.

## 4. Material and methods

### 4.1. Preparation of the casts

**Figure 7.**
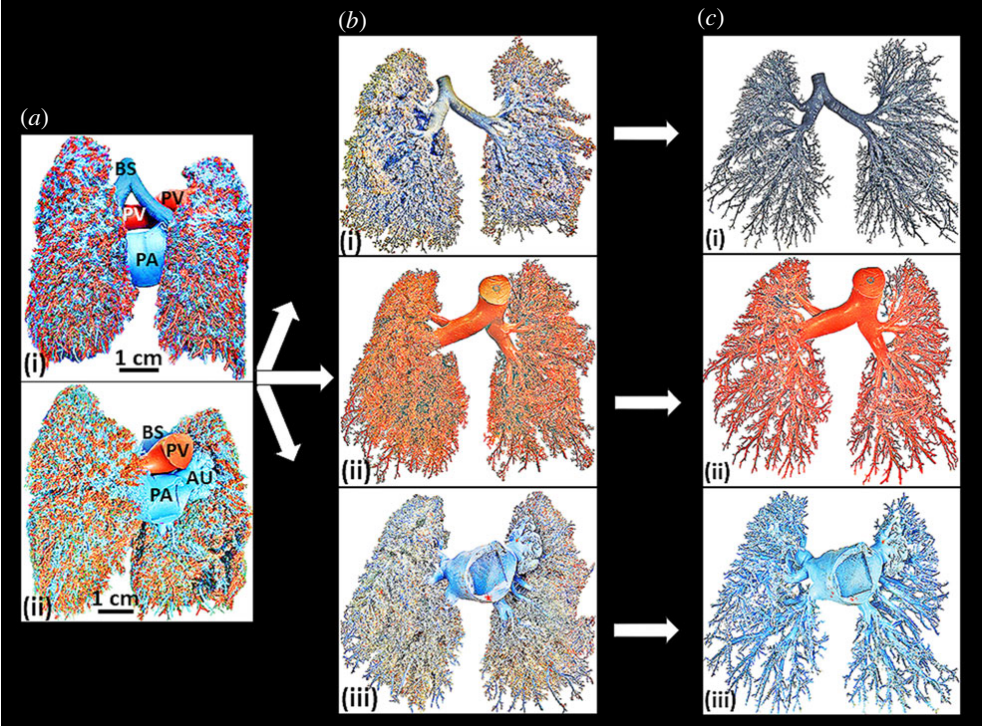
Preparation of the triple latex cast of the human lung on which measurements of diameters, lengths and angles of bifurcation were made. (*a*) Dorsal (i) and ventral (ii) views of the corroded cast of the human lung. BS (coloured grey), bronchial system; PA (coloured cyan), pulmonary artery; PV (coloured red), pulmonary vein. Au, auricle. (*b*) Separated bronchia and vascular systems. (i) Bronchial system which transports air; (ii) pulmonary vein system, which returns oxygenated blood from the lung to the heart; (iii) pulmonary artery sysem, which transports deoxygenated blood from the heart to the lung. (*c*) Pruned (cleared) casts of the bronchial and the vascular systems of the human lung. (i) Bronchial system; (ii) pulmonary vein; (iii) pulmonary artery.

As part of the programme of procuring human cadavers for teaching, a body of a 49-year-old male donor was obtained as soon as possible after death, which occurred from severe head injuries. On receiving it, the body was placed in a cold room for 1 h in ventral recumbency on a table inclined at an angle of 45°, with the head in the lower position for fluids and discharges in the airways to drain from the lungs by gravity. Any remaining materials were physically aspirated. To expose and examine the heart and the lungs to check for any damages and pathologies, after identifying the relevant anatomical landmarks, a median longitudinal incision was made with a bone cutter from the xiphoid process of the sternum, through its body to the jugular (suprasternal) notch of the manubrium. Using surgical retractors, the incision was expanded and the lungs and the heart examined after clearing any obstructing connecting tissues. While the general health and lifestyle habits (e.g. smoking) of the individual were unknown, except for small diffuse black spots that are characteristic of lungs of urban dwellers, no abnormalities, pathologies and physical injuries to the lungs and the heart were observed. The neck was extended at the atlanto-axial joint and an anteromedian incision made on the neck terminating on the suprasternal notch. The trachea was exposed and cannulated after making a transverse incision between the cricoid cartilage of the larynx and the first tracheal cartilage. The casts of the bronchial system, the pulmonary artery and the pulmonary vein were prepared with the lung *in situ* (i.e. intact in the thoracic cavity). A modification of the methods of Nelson & Manchester [30], Maina & van Gils [70], Huang *et al.* [73], König *et al.* [200] and Phalen *et al.* [201] was used to cast the human lung, which was gently inflated with air to completely fill the thoracic cavity and the pressure held at a constant pressure of 10 mbar. The pulmonary artery and vein were then identified and cannulated, and to establish vascular continuity across the pulmonary vasculature the lung was perfused at a pressure of 30 mbar above the highest point of the chest with degassed physiological saline into which heparin solution was added to promote dispersion of any blood clots in the blood vessels. The process was continued until the fluid running out of the lung (through pulmonary vein) and emptying into the left atrium of the heart run out clear. Stock solution of latex rubber, which is white in colour, was dyed dark brown for injection into the airways (figures 1 and 7), red for the pulmonary vein (figures 2 and 7) and cyan for the pulmonary artery (figures 3 and 7). The solution was well stirred for the dye to disperse evenly and then left to stand for the air bubbles to break up and/or float to the top. To hasten the process, the large bubbles were physically broken with a glass stirrer. Syringes (50 cm^3^) were filled up with the latex rubber and connected to cannulas that were attached to manometers at a T-junction. For the blood vessel, the injections were made slowly and simultaneously at a pressure of 30 mbar until the surface of the lung tensed. The blood vessels were then ligated ahead of the cannulae to keep the latex in place (i.e. inside of the particular system of the lung). Injection of latex rubber into the airways followed that of the blood vessels. The injection was done at a pressure of 10 mbar and was continued until the organs completely filled the thoracic cavity. On completion, the trachea was ligated to keep the latex in the airways. With the cast lungs in the thoracic cavity, the body was placed in a cold room for 2 days for the latex to set. Next, the heart and lungs were carefully removed from the thoracic cavity and the organs separated. The lungs were then immersed in a 10% concentration solution of potassium hydroxide (KOH) in a large plastic container and turned twice every day for one week. Thereafter, the cast was transferred into fresh 15% KOH and turned several times per day for 3 days. The extent of maceration was constantly assessed and any large adhering tissues manually removed. When the cast was well corroded, it was rinsed in running water for 1 day and then suspended in air at room temperature to dry. The quality of the cast was assessed by examining the terminal parts of the bronchial and the vascular systems, most of which detached from the cast during the physical separation and clearing the parts to expose the branches for measurements to be made. A zoom stereo light microscope and a scanning electron microscope (figure 4*a–d*) were used to examine the structures. Here, the bronchial circulation was not cast, nor was it physically isolated from the rest of the pulmonary vasculature during casting. The structural parts that formed the bronchial circulation should have been corroded away during the preparation of the cast.

### 4.2. Ordering and measuring of the branches

The bronchial, pulmonary artery and pulmonary vein systems of the cast lungs were carefully manually separated into the different parts, which were painstakingly cleared using soft plastic tweezers to expose the branches (figures 1–3 and 7). The morphogenetic, dichotomy or Weibel's ordering methods [42,63–65,73,93,95] were used to classify the branches from the trachea outwards (figure 6*a*,*b*). To ensure that the branches were not measured twice, they were numbered according to a binary system suggested by Weibel & Gomez [64] and modified by Phalen *et al.* [201]: a code was allocated to each branch, beginning with a designated letter i1 (figure 5*b*). The codes were based on those assigned to the parent branch, with the daughter branches being labelled in numerical order from left to right.

### 4.3. Measurement of the lengths, diameters and angles of bifurcation

**Figure 8.**
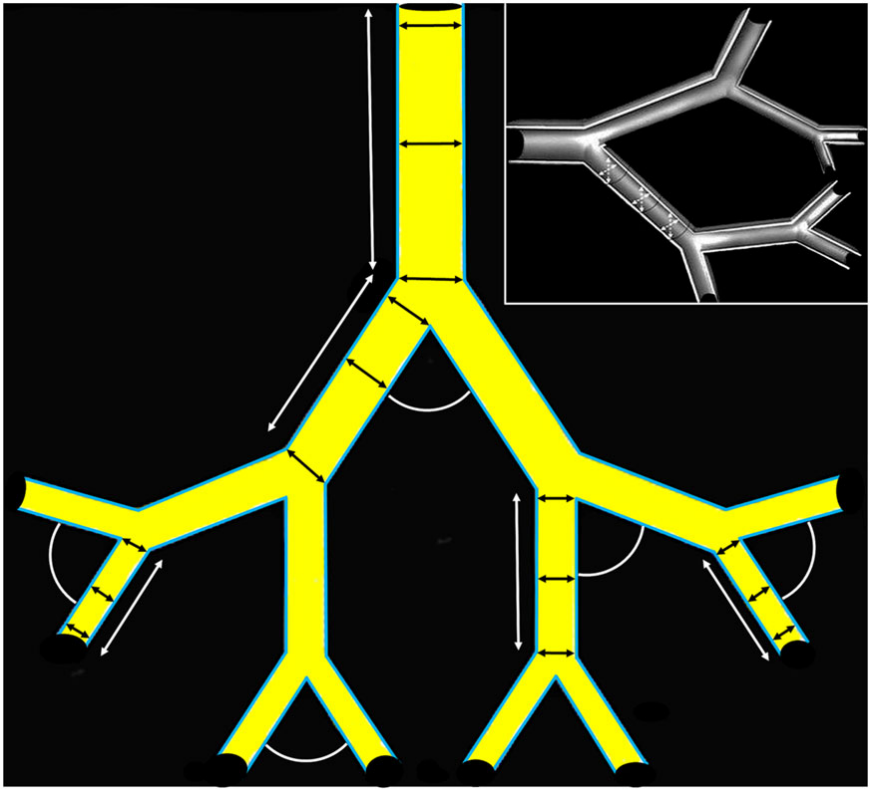
Measurement of the diameters, the lengths and the angles of bifurcation of the branches of casts of the bronchial system, the pulmonary artery and the pulmonary vein of a human lung. For the diameters (short double-sided arrows in the lumen) and the lengths (long double-sided arrows outside the lumen) measurements were made with a digital vernier calliper and the angles of bifurcation (arcs) determined by a protractor. Three measurements of the diameters (as shown) and the lengths were made and the mean value calculated. Also, for the angles, in each case, three measurements were made. Insert: View of a branched structure, with the dashed crossing arrows indicating the assumption that the cross-sectional profiles of the branches were circular (round).

The diameters and the lengths were measured by a digital vernier calliper, and the branching angles, which comprised those angles normal to the direction of gravity in a human being standing erect and those inclined at an angle to it, were measured using a protractor. Where the angles were too small and difficult to measure directly on the casts, mostly those of the terminal branches, the angles were traced on paper and measurements made of the traces with a protractor. On assumption that the cross-sectional profiles of the branches of the cast airways and the blood vessels were round and straight, for the diameters, three equally spaced measurements were made at the middle, proximal and distal bifurcation points, the length measurements were made between the bifurcation points, and the angles of bifurcation were determined at the points where branches converged (figure 8). The measurements taken in this study and used for calculation of the *D*_F_ values of the bronchial and vascular systems of the lung were made the same way.

The considerable amount of work involved in the analysis of the casts was made over a period of approximately 6 years mainly by three individuals, all of whom were knowledgeable about the structure (anatomy) of the human lung. To make certain that the measurements were accurate and reproducible, they were taken independently (by the investigators) and where discrepancies of more than 5% occurred, the measurements were rechecked and reconciled. The mean values were calculated from those determined and decided on by the three individuals. The lowest branches of the cleared bronchial and vascular systems were as follows: approximately 1 mm in diameter for the terminal bronchioles, and 0.5–0.8 mm in diameter for the arterioles and the venules.

### 4.4. Determination of the fractal dimensions (*D*_F_)

The mean diameters and lengths falling into a generation were averaged out and the values used to calculate the *D*_F_ values after plotting the data on double logarithmic axes (figures 1–3). The *D*_F_ values were determined as 1 minus the slopes of the regression lines [1,28,30,73,92], which were expressed in the format *y* = *aX^ω^*, where *a* is the *y* intercept and *ω* is the slope of the regression line. The 95% confidence intervals were added to regression lines to show the dispersion of the data points.

## Data accessibility

This article has no additional data.
